# 46, XY Complete Gonadal Dysgenesis (Swyer Syndrome) Presenting as Primary Amenorrhea in a Normomorphic Adult Female From Kakamega, Kenya

**DOI:** 10.1002/ccr3.70140

**Published:** 2025-01-20

**Authors:** Christian Omoaghe

**Affiliations:** ^1^ Department of Medicine M.P. Shah Hospital Parklands Nairobi Kenya

**Keywords:** 46 XY complete gonadal dysgenesis, gonadectomy, hormone replacement therapy, primary amenorrhea, Swyer syndrome

## Abstract

Differences/disorders of sex development (DSDs) are a diverse group of congenital conditions that result in disagreement between an individual's sex chromosomes, gonads, and/or anatomical sex. The 46, XY DSD group is vast and includes various conditions caused by genetic variants, hormonal imbalances, or abnormal sensitivity to testicular hormones, leading to varying degrees of under‐virilization. A 19‐year‐old phenotypically normal female from Kakamega, Kenya, presented with primary amenorrhea. Physical examination revealed Tanner stage 3 breast development, Tanner stage 4 pubic hair, normal external genitalia, and a gynoid body shape. Hormonal profile tests indicated hypergonadotropic hypogonadism with normal 17‐hydroxyprogesterone and testosterone levels. MRI revealed a hypoplastic uterus and absent ovaries. Karyotyping confirmed a 46, XY genotype, leading to the diagnosis of 46, XY complete gonadal dysgenesis (Swyer syndrome). Swyer syndrome is a rare disorder of sex development, characterized by unambiguous female genitalia, bilateral streak gonads, and elevated gonadotropin levels in individuals with a 46, XY karyotype. The condition results from abnormal gonadal development due to mutations in testis‐determining factors, most commonly the SRY gene. Patients typically present with primary amenorrhea and seldom have secondary sexual characteristics as this patient had. Management includes hormone replacement therapy and gonadectomy because of the increased risk of gonadal tumors. The patient was educated about her condition, initiated on combined contraceptive pills, and counseled on exploratory laparoscopic gonadectomy. This case highlights the importance of a comprehensive diagnostic approach in patients with primary amenorrhea, keeping in mind that patients with disorders of sex development may have developed secondary sexual characteristics.

**Summary**

- Delayed menstruation is the most prevalent initial symptom among individuals with 46, XY disorder of sex development (DSD) and adolescent females with this rare disorder may have secondary sexual characteristics.

## 1 Introduction

Differences/disorders of sex development (DSDs) are a diverse group of congenital conditions that result in disagreement between an individual's sex chromosomes, gonads, and/or anatomical sex. The 46, XY DSD group is vast and includes various conditions caused by genetic variants, hormonal imbalances, or abnormal sensitivity to testicular hormones, leading to varying degrees of under‐virilization. The term 46, XY DSD females refers to phenotypic females with a male genotype, previously referred to as sex reversal, intersex, or pseudohermaphroditism. 46, XY DSD can be divided into two main categories: (1) disorders of sex determination involving abnormal gonadal development, and (2) disorders of sex differentiation involving altered production of testicular hormones or altered peripheral response to steroid or protein hormones produced by the fetal testis (androgen insensitivity syndrome [AIS]) [1].

### 1.1 46, XY DSD Characterized by Abnormal Gonadal Development

Gonadal dysgenesis can be caused by mutations in testes determining factors on the Y chromosome. In approximately 10%–20% of cases, a mutation is found in the sex‐determining region Y (SRY) gene [2]. A number of patients with 46, XY gonadal dysgenesis have been reported with a duplication of the Xp21.2 → p22.11 region of the X chromosome, which contains the DAX1 gene [3]. Inheritance patterns that have been reported include sporadic cases (de novo variants), autosomal dominant, autosomal recessive, X‐linked recessive, and Y‐linked. Individuals with pure 46, XY gonadal dysgenesis typically present normal external female genitalia. The gonads are streak gonads without hormone production, so Müllerian structures are present, but no development of secondary female characteristics during puberty is typically observed [2]. Adrenarche is usually normal. The completely undeveloped streak gonads are associated with a 15%–30% increased risk of abdominal tumors, most commonly dysgerminoma [1, 4]. Elevated gonadotropin levels and low testosterone and dihydrotestosterone levels are typically observed in these individuals [5].

### 1.2 46, XY DSD Characterized by Androgen Insensitivity

AIS stems from X‐linked androgen receptor mutations, which hamper androgen‐dependent male sexual differentiation to varying extents. Clinically, individuals with complete AIS exhibit female external genitalia. Although the gonads have differentiated into testes, they can be found in different locations such as the intra‐abdominal region, inguinal canals, or within the labia. The regression of Müllerian structures (i.e., the upper part of the vagina, uterus, and Fallopian tubes) occurs due to the normal production of anti‐Müllerian hormone in the fetus. During puberty, breast development occurs as a result of the aromatization of excess androgens into estrogens, although pubic and axillary hair are usually scarce or absent. In contrast, individuals with partial AIS exhibit varying degrees of virilization at birth. Consequently, these patients may be raised as either females or males. Elevated gonadotropin levels and normal or elevated testosterone levels are common in these patients [2, 5].

46, XY females are an uncommon occurrence, with limited data available on their incidence. Estimates of the prevalence of AIS and gonadal dysgenesis range widely, from 1 to 5 per 100,000 births [6] and 1 per 80,000 births [7], respectively. AIS is considered to be the most common cause of 46, XY DSD, followed by gonadal dysgenesis.

Ultrasound is generally recommended as the first imaging method for clinical evaluation because of its accessibility and the lack of necessity for sedation or contrast agents. Nevertheless, the quality of information provided by ultrasound relies on the expertise of the evaluator and the device utilized. Magnetic resonance imaging (MRI) is better suited for visualizing gonads, even though streak gonads can be difficult to detect using MRI. Laparoscopy is the gold standard for evaluating these intra‐abdominal tissues [5].

## 2 Case Presentation

### 2.1 Case History/Examination

A 19‐year‐old woman presented to the outpatient clinic in January 2024 with a failure to attain menarche. Her developmental milestones were attained at appropriate ages, and she had been developing phenotypically. She had not commenced sexual activity. She was the second child of four children (two males and two females), her younger sister being 10 years old, and developing well so far. Her mother who often accompanied her to the clinic was tall (179 cm), had attained menarche at the age of 14 years, and looked phenotypically normal. There was no family history of genital ambiguity. The patient was tall (height 177 cm), weighed 53 kg, and had an upper‐to‐lower segment ratio of 1:1. Her waist circumference was 88 cm, hip circumference was 103 cm and had a feminine voice with sparse axillary hair. The patient had palpable breasts (Tanner stage 3) and pubic hairs (Tanner stage 4). Her external genitalia appeared normal, although the vaginal length was not determined because her mother declined consent.

### 2.2 Investigations

Hormonal profile tests revealed hypergonadotropic hypogonadism and normal 17‐hydroxyprogesterone and testosterone levels (Table 1). Anti‐Mullerian hormone level was not measured. An ultrasound scan of the abdomen and pelvis revealed a normal, anteflexed uterus with a normal endometrial stripe and no evidence of ovarian pathology. However, the sonographic findings were inconsistent with the hormonal profile. She was advised to undergo an MRI scan of the pelvis, which revealed a hypoplastic uterus with absent ovaries. She subsequently underwent karyotyping, which revealed karyotype 46, XY.

**TABLE 1:** Hormonal profile of patient showing hypergonadotropic hypogonadism.

Test	Result	Units	Status	Normal range
17 beta estradiol	< 11.80	pg/mL		
FSH	274.00 mIU/mL Comments: reference ranges: follicular phase…0.3.5–12.5 mIU/mL			
17 hydroxy progesterone	0.57	ng/mL		
Prolactin	14.69	ng/mL	*N*	2.1–18.4
Testosterone	0.14	ng/mL	*N*	0.06–0.82
Luteinizing hormone	35.03 mIU/mL Comments: adult female (follicular phase…2.4–12.6 mIU/mL)			
Free triiodothyronine (FT3)	3.43 pg/mL Comments: in case of females: females on contraception: 2.6–4.5 pg/mL		*N*	2–4.4
Free thyroxine (FT4)	1.45 ng/mL Comments: in case of females: pregnant women 1st trimester: 0.9–1.5 ng/mL		*N*	0.93–1.97
Thyroid stimulating hormone (TSH)	1.87	μU/mL	*N*	0.51–4.3

### 2.3 Differential Diagnosis

A diagnosis of primary amenorrhea secondary to 46, XY (complete gonadal agenesis) was made, and the patient and her mother were educated about the medical condition, its prognosis, and fertility prospects in the future.

### 2.4 Treatment

She was started on a combined contraceptive pill (Femiplan) which contains 0.03 mg of ethinylestradiol and 0.15 mg of levonorgestrel and experienced withdrawal bleeding in the third cycle.

### 2.5 Follow‐Up

She has been counseled on exploratory laparoscopic gonadectomy but has not yet consented.

## 3 Discussion

The term 46, XY DSD females refers to phenotypic females with a male genotype and may be divided into two broad categories: (1) disorders of sex determination characterized by abnormal gonadal development and (2) disorders of sex differentiation characterized by altered production of testicular hormones or altered peripheral response to steroid or protein hormones produced by the fetal testis (AIS). Swyer syndrome is a rare disorder of sex development, characterized by unambiguous female genitalia, bilateral streak gonads, and elevated gonadotropin levels in individuals with a 46, XY karyotype who typically present with delayed puberty, primary amenorrhea and a lack of secondary sexual characteristics [3, 8]. This young woman had 46, XY features with abnormal gonadal development (complete gonadal agenesis). The absence of ambiguous genitalia in this patient and her normal testosterone and 17‐hydroxyprogesterone levels ruled out congenital adrenal hyperplasia and other rare genetic syndromes. Other causes of 46, XY DSD are shown in Table 2 [3]. Swyer syndrome can present with serum testosterone within normal limits [9]. Her pedigree suggested that the mutation was a de novo mutation, although genetic studies of her parents and siblings would be valuable in confirming the genetic basis of her condition. The lack of these further genetic studies is a limitation of this report. The patient had a gynoid body shape and her height may have been familial (her mother's height was 179 cm). The gynoid pattern of fat distribution is influenced by hormones, such as estrogen and progesterone, which promote the accumulation of fat in the hips, thighs, and buttocks. Adrenarche and aromatization of androgens to estrogens might have been responsible for the feminine features of this patient despite a very low level of β‐estradiol, though the presence of a hypoplastic uterus might be a result of the low estrogen [2]. The chronic hypoestrogenic environment may have led to an upregulation of estrogen receptors in breast and adipose tissue thereby increasing their sensitivity to the estrogen produced by peripheral conversion from androgens. There are also reports of possible production of female sex hormones by the streak gonads [10, 11]. Laparoscopy with prophylactic gonadectomy is recommended for these patients even before the commencement of estrogen replacement [3]. This is because of the difficulty in detecting streak gonads using MRI [5]. However, the patient's age at presentation (19 years) seemed postpubertal, which implies years of hypoestrogenemia with implications for her bone mineral density (increased risk of osteoporosis). The presence of a Y chromosome in the genome has been implicated as the cause of the increased risk of dysgerminoma and gonadoblastoma in these patients and not hormone replacement therapy [1, 3, 4, 5].

**TABLE 2:** Causes of 46 XY, DSD.

Disorders of gonadal (testis) development	Complete or partial gonadal dysgenesis (due to genetic variants in SRY, SOX9, NRSA1, WT1, DHH, DMRT1, etc.)
	Ovotesticular DSD
	Testis regression
Disorders of androgen synthesis	LH receptor mutations
	Smith–Lemli–Opitz syndrome
	Steroidogenic acute regulatory protein mutations*
	Cholesterol side chain cleavage (CYP1IA1)*
	3β‐hydroxysteroid dehydrogenase 2 (HSD3B2)*
	17α‐hydroxylase/17,20‐lyase (CYP17)*
	P450 oxidoreductase (POR)
	17β‐hydroxysteroid dehydrogenase (HSD17B3)
	5α‐reductase2 (SRD5A2)
Disorders of androgen action	Androgen insensitivity syndrome (complete, partial, and minimal)
	Drugs and environmental modulators of androgen receptor activity
Disorders of AMH synthesis or action	Persistent mullerian duct syndrome
Other	Syndromic associations of male genital development (e.g., cloacal anomalies, Robinow, Aarskog, and hand‐foot‐genital syndromes)
	Vanishing testis syndrome
	Isolated hypospadias (CXorf6)
	Congenital hypogonadotropic hypogonadism
	Cryptorchidism (INSL3, GREAT)
	Environmental endocrine disruptors

* Associated with congenital adrenal hyperplasia.

## 4 Conclusions

Phenotypic female 46, XY DSD is a rare disorder that is most often diagnosed in adolescence. This case highlights the need to consider 46, XY DSD in the setting of primary amenorrhea even when pubertal development is present. Normal breast development may mask gonadal dysgenesis and delay diagnosis. Early sex hormone treatment helps to induce and maintain typical pubertal and psychosexual development and achieve an optimal peak bone mass.

## Author Contributions

**Christian Omoaghe:** conceptualization, data curation, formal analysis, funding acquisition, investigation, methodology, project administration, resources, supervision, validation, visualization, writing – original draft, writing – review and editing.

## Consent

Written informed consent was obtained from the patient for publication of this case report and any accompanying images. A copy of the written consent is available for review by the Editor‐in‐Chief of this journal.

## Conflicts of Interest

The author declares no conflicts of interest.

## Data Availability

Data available on request from the authors.
